# Supplementary material for: Associations between plasma nucleoside reverse transcriptase inhibitors concentrations and cognitive function in people with HIV
Source: PLoS One. 2021 Jul 21;16(7):e0253861. doi: 10.1371/journal.pone.0253861 (PMC8294567; doi:10.1371/journal.pone.0253861)

**Associations between plasma nucleoside reverse transcriptase inhibitors concentrations and cognitive function in people with HIV**

**Supplementary Material**

Davide De Francesco (1), Xinzhu Wang (2), Laura Dickinson (3), Jonathan Underwood (2, 4), Emmanouil Bagkeris (1), Daphne S. Babalis (5), Patrick W. G. Mallon (6), Frank A. Post (7), Jaime H. Vera (8), Memory Sachikonye (9), Ian Williams (1), Saye Khoo (3), Caroline A. Sabin (1), Alan Winston (2) and Marta Boffito (2, 10) on behalf of the Pharmacokinetic and Clinical Observations in PeoPle Over fiftY (POPPY) study

1. Institute for Global Health, University College London, London, UK;
2. Department of Infectious Disease, Imperial College London, London, UK;
3. Department of Molecular & Clinical Pharmacology, University of Liverpool, Liverpool, UK;
4. Division of Infection and Immunity, University of Cardiff, Cardiff, UK;
5. Imperial Clinical Trials Unit, Imperial College London, London, UK;
6. University College Dublin School of Medicine, Dublin, Ireland;
7. King's College Hospital NHS Foundation Trust, London, UK;
8. Department of Global Health and Infection, Brighton and Sussex Medical School, Brighton, UK;
9. UK Community Advisory Board, London, UK;
10. Chelsea and Westminster Healthcare NHS Foundation Trust, London, UK;

**Supplementary Table 1:** Effect size (Eta^2^) for the association between each potential confounder and cognitive score

| **Variable** | **Global Z** | | **Visual Learning** | | **Psychomotor** | | **Visual Attention** | | **Executive Function** | | **Verbal Learning** | | **Working Memory** | |
| --- | --- | --- | --- | --- | --- | --- | --- | --- | --- | --- | --- | --- | --- | --- |
|  | **Eta^2^** | **p-value** | **Eta^2^** | **p-value** | **Eta^2^** | **p-value** | **Eta^2^** | **p-value** | **Eta^2^** | **p-value** | **Eta^2^** | **p-value** | **Eta^2^** | **p-value** |
| **Gender** | 0.029 | <.0001 | 0.005 | 0.10 | 0.026 | 0.0002 | 0.036 | <.0001 | 0.006 | 0.07 | 0.000 | 0.67 | 0.037 | <.0001 |
| **Ethnicity** | 0.096 | <.0001 | 0.050 | <.0001 | 0.051 | <.0001 | 0.031 | <.0001 | 0.046 | <.0001 | 0.021 | 0.0006 | 0.065 | <.0001 |
| **Education** | 0.079 | <.0001 | 0.064 | <.0001 | 0.050 | <.0001 | 0.016 | 0.11 | 0.028 | 0.009 | 0.045 | 0.0001 | 0.034 | 0.002 |
| **Alcohol consumption** | 0.038 | <.0001 | 0.037 | <.0001 | 0.013 | 0.03 | 0.015 | 0.02 | 0.027 | 0.0006 | 0.009 | 0.09 | 0.029 | 0.0003 |
| **Recreational drug use** | 0.019 | 0.001 | 0.008 | 0.04 | 0.008 | 0.04 | 0.017 | 0.002 | 0.017 | 0.002 | 0.000 | 0.74 | 0.015 | 0.005 |
| **Use of boosted PI** | 0.000 | 0.84 | 0.000 | 0.80 | 0.000 | 0.68 | 0.000 | 0.81 | 0.001 | 0.40 | 0.000 | 0.88 | 0.000 | 0.69 |
| **Use of Efavirenz** | 0.003 | 0.23 | 0.002 | 0.31 | 0.000 | 0.94 | 0.000 | 0.86 | 0.004 | 0.16 | 0.003 | 0.18 | 0.001 | 0.39 |
| **Age** | 0.005 | <.0001 | 0.026 | <.0001 | 0.036 | <.0001 | 0.006 | <.0001 | 0.000 | <.0001 | 0.037 | <.0001 | 0.109 | <.0001 |
| **BMI** | 0.050 | 0.003 | 0.051 | 0.02 | 0.031 | 0.02 | 0.046 | 0.004 | 0.021 | 0.09 | 0.065 | 0.66 | 0.016 | 0.03 |
| **eGFR** | 0.064 | 0.55 | 0.050 | 0.83 | 0.016 | 0.89 | 0.028 | 0.63 | 0.045 | 0.96 | 0.034 | 0.22 | 0.001 | 0.34 |
| **PHQ-9 score** | 0.037 | <.0001 | 0.013 | 0.03 | 0.015 | 0.0002 | 0.027 | <.0001 | 0.009 | 0.40 | 0.029 | 0.001 | 0.036 | 0.04 |

BMI: body-mass index; PI: protease inhibitor; eGFR: estimated glomerular filtration rate; PHQ-9: depressive symptom score from the Patient Health Questionnaire-9

**Supplementary Table 2:** Effect size (Eta^2^) for the association between each potential confounder and 3TC and ABC PK parameters

| **Variable** | **3TC AUC_0-24_** | | **3TC C_max_** | | **3TC C_trough_** | | **ABC AUC_0-24_** | | **ABC C_max_** | | **ABC C_trough_** | |
| --- | --- | --- | --- | --- | --- | --- | --- | --- | --- | --- | --- | --- |
|  | **Eta^2^** | **p-value** | **Eta^2^** | **p-value** | **Eta^2^** | **p-value** | **Eta^2^** | **p-value** | **Eta^2^** | **p-value** | **Eta^2^** | **p-value** |
| **Gender** | 0.001 | 0.77 | 0.015 | 0.27 | 0.002 | 0.68 | 0.036 | 0.09 | 0.034 | 0.10 | 0.001 | 0.81 |
| **Ethnicity** | 0.016 | 0.25 | 0.007 | 0.44 | 0.021 | 0.19 | 0.011 | 0.33 | 0.025 | 0.15 | 0.002 | 0.72 |
| **Education** | 0.068 | 0.35 | 0.035 | 0.73 | 0.084 | 0.22 | 0.071 | 0.32 | 0.033 | 0.76 | 0.114 | 0.09 |
| **Alcohol consumption** | 0.028 | 0.31 | 0.016 | 0.53 | 0.005 | 0.82 | 0.002 | 0.90 | 0.000 | 1.00 | 0.008 | 0.72 |
| **Recreational drug use** | 0.019 | 0.21 | 0.014 | 0.28 | 0.014 | 0.29 | 0.027 | 0.14 | 0.009 | 0.40 | 0.010 | 0.37 |
| **Use of boosted PI** | 0.048 | 0.04 | 0.021 | 0.19 | 0.039 | 0.07 | 0.053 | 0.03 | 0.008 | 0.41 | 0.059 | 0.03 |
| **Use of Efavirenz** | 0.040 | 0.07 | 0.008 | 0.42 | 0.041 | 0.06 | 0.016 | 0.25 | 0.000 | 0.96 | 0.040 | 0.07 |
| **Age** | 0.123 | 0.001 | 0.022 | 0.18 | 0.101 | 0.003 | 0.005 | 0.54 | 0.017 | 0.23 | 0.069 | 0.02 |
| **BMI** | 0.010 | 0.37 | 0.015 | 0.27 | 0.002 | 0.70 | 0.010 | 0.37 | 0.014 | 0.29 | 0.000 | 0.89 |
| **eGFR** | 0.296 | <.0001 | 0.018 | 0.24 | 0.230 | <.0001 | 0.039 | 0.08 | 0.000 | 0.97 | 0.089 | 0.01 |
| **PHQ-9 score** | 0.013 | 0.32 | 0.002 | 0.67 | 0.015 | 0.28 | 0.000 | 0.95 | 0.001 | 0.81 | 0.000 | 0.90 |

BMI: body-mass index; PI: protease inhibitor; eGFR: estimated glomerular filtration rate; PHQ-9: depressive symptom score from the Patient Health Questionnaire-9

**Supplementary Table 3:** Effect size (Eta^2^) for the association between each potential confounder and FTC and TDF PK parameters

| **Variable** | **FTC AUC_0-24_** | | **FTC C_max_** | | **FTC C_trough_** | | **TDF AUC_0-24_** | | **TDF C_max_** | | **TDF C_trough_** | |
| --- | --- | --- | --- | --- | --- | --- | --- | --- | --- | --- | --- | --- |
|  | **Eta^2^** | **p-value** | **Eta^2^** | **p-value** | **Eta^2^** | **p-value** | **Eta^2^** | **p-value** | **Eta^2^** | **p-value** | **Eta^2^** | **p-value** |
| **Gender** | 0.000 | 0.88 | 0.000 | 0.99 | 0.000 | 0.89 | 0.009 | 0.04 | 0.033 | <.0001 | 0.001 | 0.50 |
| **Ethnicity** | 0.010 | 0.03 | 0.014 | 0.01 | 0.005 | 0.14 | 0.007 | 0.06 | 0.000 | 0.83 | 0.011 | 0.02 |
| **Education** | 0.013 | 0.32 | 0.011 | 0.39 | 0.013 | 0.32 | 0.016 | 0.17 | 0.020 | 0.08 | 0.011 | 0.39 |
| **Alcohol consumption** | 0.000 | 0.92 | 0.000 | 0.95 | 0.001 | 0.79 | 0.002 | 0.68 | 0.005 | 0.31 | 0.001 | 0.88 |
| **Recreational drug use** | 0.030 | 0.00 | 0.033 | <.0001 | 0.025 | 0.001 | 0.007 | 0.06 | 0.000 | 0.83 | 0.012 | 0.02 |
| **Use of boosted PI** | 0.008 | 0.06 | 0.008 | 0.05 | 0.005 | 0.11 | 0.004 | 0.19 | 0.002 | 0.37 | 0.004 | 0.19 |
| **Use of Efavirenz** | 0.008 | 0.05 | 0.008 | 0.05 | 0.005 | 0.11 | 0.003 | 0.20 | 0.000 | 0.70 | 0.005 | 0.11 |
| **Age** | 0.157 | <.0001 | 0.189 | <.0001 | 0.099 | <.0001 | 0.070 | <.0001 | 0.028 | 0.0003 | 0.075 | <.0001 |
| **BMI** | 0.000 | 0.98 | 0.000 | 0.94 | 0.000 | 0.98 | 0.095 | <.0001 | 0.271 | <.0001 | 0.013 | 0.01 |
| **eGFR** | 0.136 | <.0001 | 0.172 | <.0001 | 0.083 | <.0001 | 0.124 | <.0001 | 0.042 | <.0001 | 0.135 | <.0001 |
| **PHQ-9 score** | 0.004 | 0.20 | 0.002 | 0.31 | 0.004 | 0.19 | 0.001 | 0.53 | 0.001 | 0.51 | 0.002 | 0.39 |

BMI: body-mass index; PI: protease inhibitor; eGFR: estimated glomerular filtration rate; PHQ-9: depressive symptom score from the Patient Health Questionnaire-9

**Supplementary Figure 1:** Regression coefficients (i.e. adjusted rho) from rank regression evaluating the associations of PK parameters with global z-scores. Univariable estimates: one NRTI at the time in separate models; multivariable estimates: both NRTIs in the regimen in the same model (one model for each PK parameter).


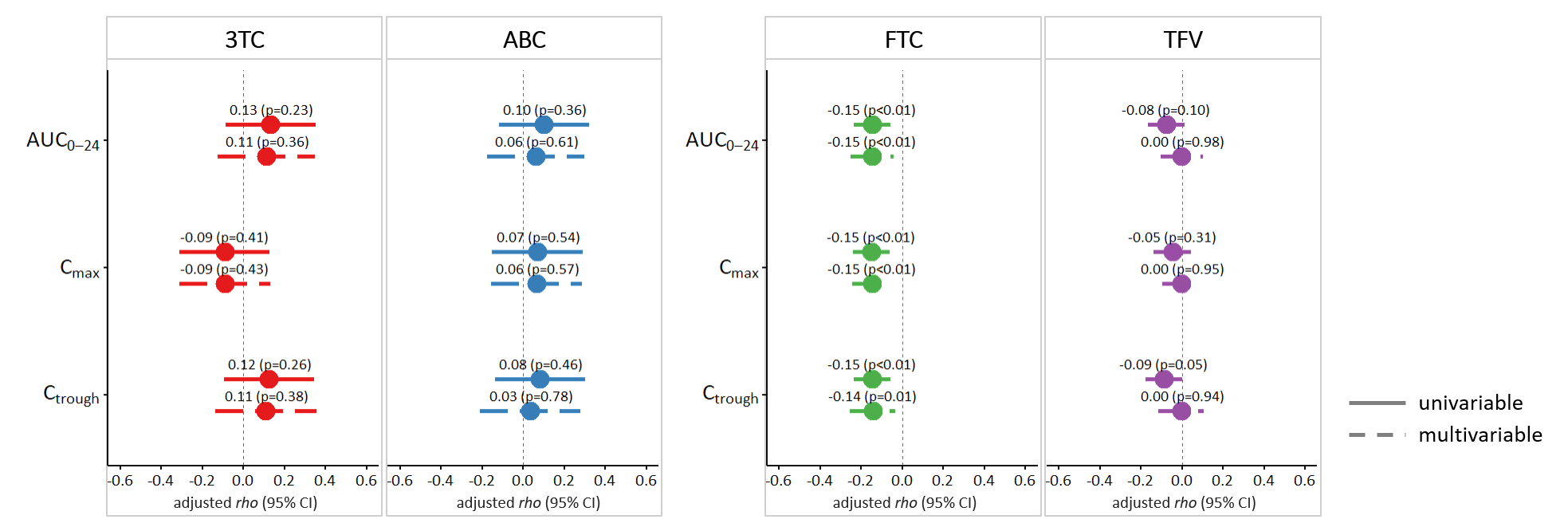


**Supplementary Figure 2:** Regression coefficients (i.e. adjusted rho) from rank regression evaluating the associations of PK parameters with domain z-scores. Univariable estimates: one NRTI at the time in separate models; multivariable estimates: both NRTIs in the regimen in the same model (one model for each PK parameter).


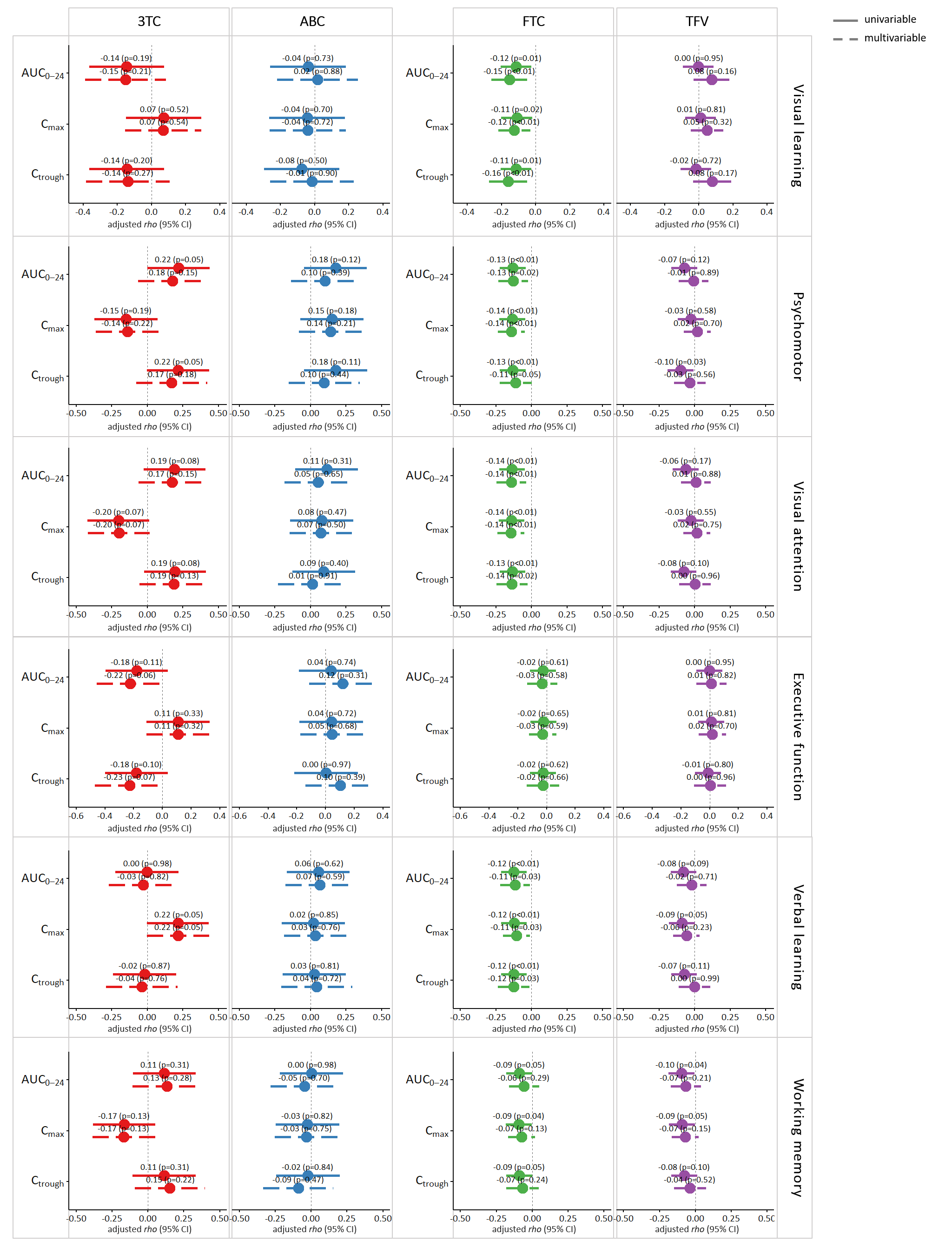


**Supplementary Figure 3:** Regression coefficients (i.e. adjusted rho) from rank regression evaluating the associations of PK parameters with domain z-scores. Associations are adjusted for age, gender, ethnicity, education, estimated glomerular filtration rate, use of ritonavir/cobicistat boosted protease inhibitor and use of efavirenz (3TC and ABC PK parameters), plus BMI (for FTC and TFV PK parameters only). Univariable estimates: one NRTI at the time in separate models; multivariable estimates: both NRTIs in the regimen in the same model (one model for each PK parameter).


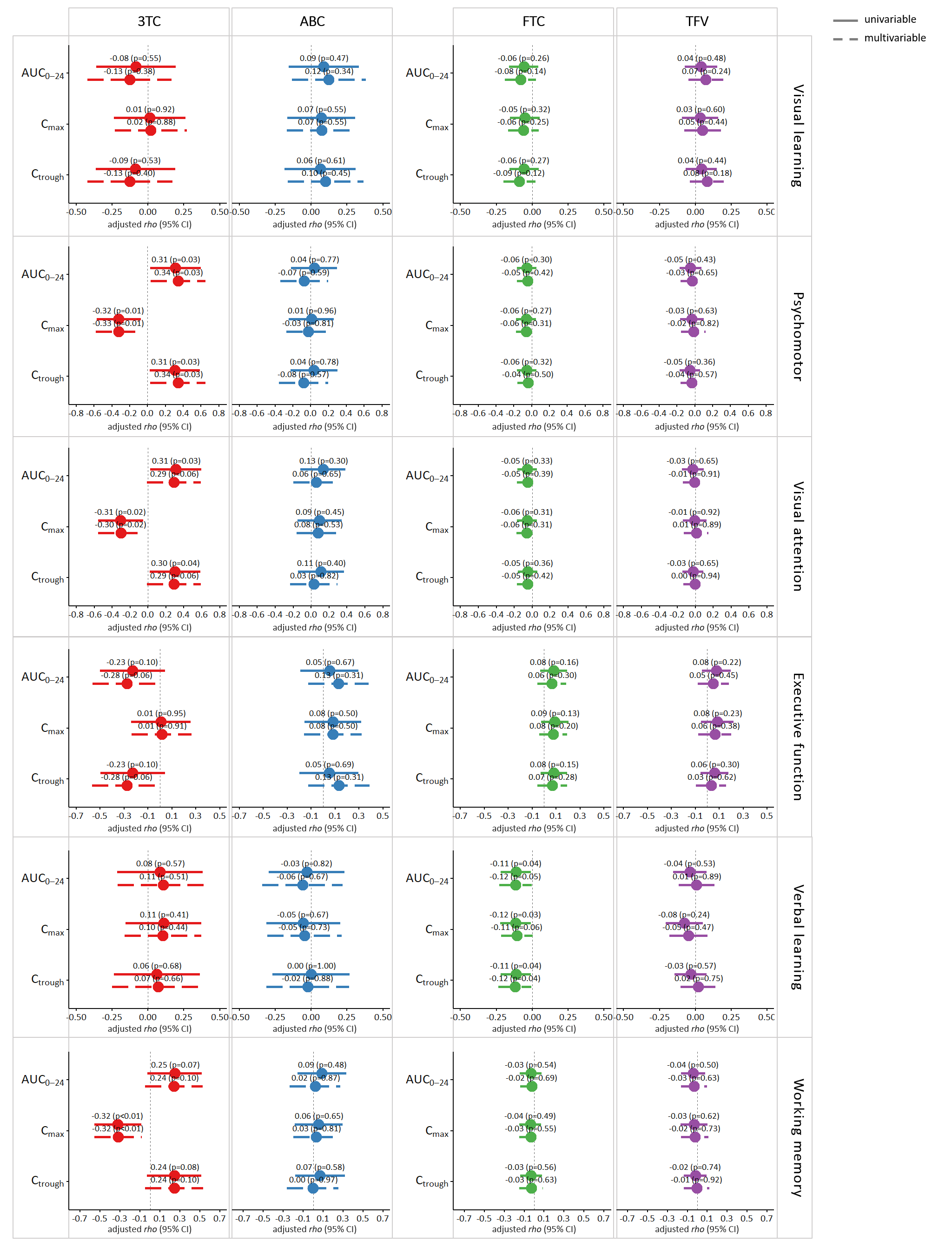

Supplement: S1 File — (DOC) [file pone.0253861.s001.doc]
